# Car2Car Communication Using a Modulated Backscatter and Automotive FMCW Radar

**DOI:** 10.3390/s21113656

**Published:** 2021-05-24

**Authors:** Antonio Lazaro, Marc Lazaro, Ramon Villarino, David Girbau, Pedro de Paco

**Affiliations:** 1 Department of Electronics, Electrics and Automatic Control Engineering, Rovira i Virgili University, 43007 Tarragona, Spain; marc.lazaro@urv.cat (M.L.); ramon.villarino@urv.cat (R.V.); david.girbau@urv.cat (D.G.); 2 Telecommunications and Systems Engineering Department, Universitat Autonoma de Barcelona (UAB), 08193 Bellaterra, Spain; pedro.depaco@uab.cat

**Keywords:** Car2Car communications, V2X, automotive radar, FMCW radar, backscatter, RFID

## Abstract

This work proposes the use of a modulated tag for direct communication between two vehicles using as a carrier the wave emitted by an FMCW radar installed in the vehicle for advanced driver assistance. The system allows for real-time signals detection and classification, such as stop signal, turn signals and emergency lights, adding redundancy to computer video sensors and without incorporating additional communication systems. A proof-of-concept tag has been designed at the microwave frequency of 24 GHz, consisting of an amplifier connected between receiving and transmitting antennas. The modulation is performed by switching the power supply of the amplifier. The tag is installed on the rear of the car and it answers when it is illuminated by the radar by modulating the backscattered field. The information is encoded in the modulation switching rate used. Simulated and experimental results are given showing the feasibility of the proposed solution.

## 1. Introduction

Nowadays, collisions in urban environments are unfortunately too frequent [1]. The high density of vehicles, bad weather conditions, driver’s age, fatigue or distractions are some of the reasons for the high number of collisions in cities [2]. In addition to the danger posed by such collisions, they cause major mobility problems and increase air pollution. In order to reduce road fatalities, several technologies to improve vehicle safety have been adopted. Radar is a key sensor for advanced driver assistance systems (ADAS). Automotive radar is a mature technology which, due to advances in millimeter wave (mmWave) technology, is available even for mid-range cars [3]. Future generations of autonomous vehicles will need sensors to recognize the environment in real-time. Radar technology is not affected by poor weather or light conditions [4,5] in comparison with other technologies such as camera-based sensors or LIDAR. Therefore, the automotive radar market is growing and will continue, driven by safety and autonomous driving. Automotive radars are based on frequency-modulated continuous-wave radar (FMCW). FMCW radars can measure simultaneously the target range and its relative velocity.

Transmissions at mmWave bands are challenging due to a significantly higher path loss than those at lower frequencies. Despite this drawback, the interest in millimeter wave frequency bands for communication applications has recently arisen as the technology has matured. Therefore, microwave and millimeter-wave identification (MMID) systems have been investigated at these bands (24 GHz, 60 GHz and 77 GHz) [6,7,8,9]. The advantages of MMID systems are the use of smaller directive reader antennas as well as a higher bandwidth for data handling. The higher path loss caused by the frequency of operation can be mitigated with the use of readers integrating beamforming techniques and tags with retrodirective arrays [9]. The large bandwidth available at mmWave bands allows high transmission rate transfers. In addition, the advantage of MMID systems compared to other advanced communications systems is the low latency due to its light communication protocol. Moreover, readers based on FMCW radar allows to locate the tags [10,11,12,13]. Examples of miniature chipless MMID have been proposed in the literature [14,15].

In recent years, microwave and mm-wave radars have been increasingly used in automotive, industrial and communications applications. The availability of low-cost millimeter band radars has allowed the emergence of new applications such as contactless and unobtrusive vital sign measurement with a millimeter-wave radar system [16,17,18,19]. In these applications, small phase changes due to micro-Doppler chest movements are used to extract the breathing and heart rate from Doppler or FMCW radars. Recently, MIMO radars similar to those used in this work are considered for these applications [20,21]. Recently, another application to detect acoustic signals based on the micro-Doppler effect has been presented [22]. Doppler-based radars have also been applied to structural health monitoring [23].

In this context, the authors have recently presented a mechanical device based on a rotating corner that was used to indicate the position of pedestrians, damaged cars or workers on the road [24]. The combination of a corner reflector with the rotation introduces a strong return signal that can be detected by automotive FMCW radars. However, this type of device has some drawbacks such as the size and power consumption due to its mechanical construction since it uses a motor. In this work, the authors extend the idea of using modulated transponders to improve safety while driving. However, an electronic device to address these issues and expand the possible use cases is proposed. This paper introduces an approach to vehicle-to-vehicle communication (car-to-car or car2car) taking advantage of automotive radar. It proposes a system for car-to-car communications based on a backscattered tag or transponder located on in the rear of a vehicle and the FMCW radar located in the front of another vehicle. The advantage over other communication technologies is that it does not require additional communication infrastructure or the addition of new communication equipment, since the radar of the ADAS system is intended to be used as a reader [10,11,12,13]. The proposed system constitutes an alternative to those based on technologies such as computer vision, thus being a complement to other advanced wireless communication systems used in Vehicular Ad-hoc Network (VANET) [25,26,27].

The paper is organized as follows: Section 2 describes the design of the tag and its detection using an automotive FMCW radar. Experimental results are provided in Section 3. A discussion comparing different microwave and mm-wave backscatters is given in Section 4. Finally, some conclusions are included in Section 5.

## 2. Tag Design and Detection

### 2.1. System Overview

**Figure 1:**
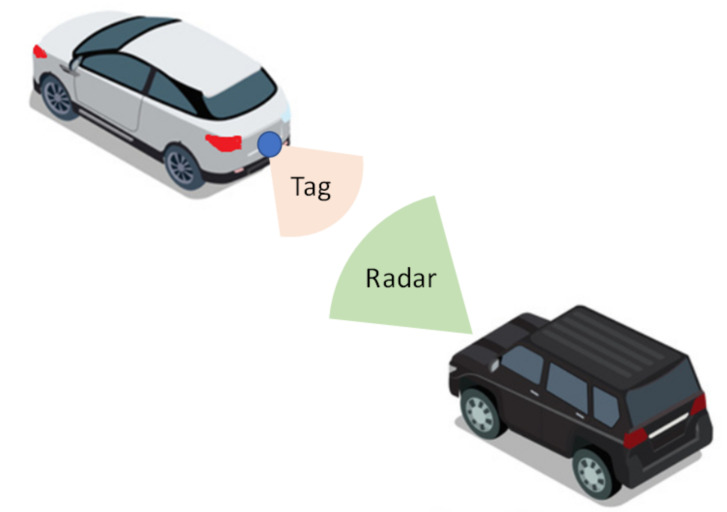
Schema of car2car communication based on a backscatter tag located at the rear of the vehicle and illuminated by the radar.

**Figure 2:**
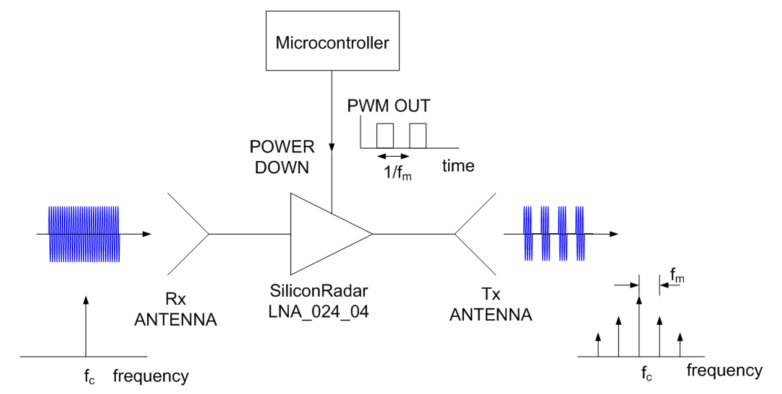
Block diagram of the modulated backscatter.

A sketch of the system with a tag installed at the rear of a car when is illuminated by an automotive radar is shown in Figure 1. The tag is connected to the electronic control unit (ECU) of the car. When the ECU activates the turn lights, the warning or the brakes, it also enables the modulation of the tag answering to the radar. A proof-of-concept tag has been designed for validation in outdoor experiments. The block diagram of the tag is shown in Figure 2. The tag is based on a modulated transponder (semi-passive tag) that backscatters the incoming signal. The radar receives this modulated signal by modifying the Doppler spectrum, thus allowing the identification of the object. It is important to note that no further modifications to the vehicle’s radar hardware are required. The tag consists of an amplifier whose input and output are connected to a pair of series-fed patch array antennas. The signal from the radar is received by the input antenna; it is amplified and retransmitted through the output antenna. The amplifier employed in this tag is a Silicon Germanium MMIC (Monolithic Microwave Integrated Circuit) amplifier LNA_24_04 from Silicon Radar GmbH. This amplifier operates in the 24 GHz to 29 GHz frequency band. Its current consumption is 5.6 mA at 3.3V and it has a typical gain of 17 dB at 24 GHz. The amplifier has a power-down control pin that is used to enable/disable the amplifier. This pin is connected to the pulse-width modulation (PWM) output of a microcontroller (ATtiny85 from Atmel). The switching rate of this digital oscillator can be configured by the microcontroller depending on the message to answer (e.g., turn light enabled, warning lights turned on, brakes activation). The antennas used in the prototype consist of a series-fed array with seven elements. Each element of the antenna is a rectangular patch. The array is designed to cover the 24–24.25 GHz ISM (Industrial, Scientific, Medical) band. This array topology has been chosen because a series-fed array requires less space than corporate-fed arrays [28]. Besides, the tapering technique that consists of changing the size of the patches to change the feed amplitude at each patch has been used. This technique allows to improve the side lobes level with respect to the array with uniform patches [29]. The antenna has been designed with Ansys HFSS.

The backscattered power is proportional to the differential radar cross section, $RCD_{dif}$ [30]:

(1) $$RCS_{dif}=\frac{\lambda^{2}}{4\pi }G_{t,in}G_{a}G_{t,out}m,$$

where λ is the wavelength, $G_{t,in}$ and $G_{t,out}$ are the transponder’s input and output antennas gain, respectively, $G_{a}$ is the amplifier gain and *m* is the modulation factor. *m* is given by the square of the Fourier coefficient at the modulation frequency offset $f_{m}$ from the central carrier. For a 50% duty cycle square waveform, it is given by $m=1/\pi^{2}$ [10]. The use of an active transponder with gain allows to increase the differential radar cross section compared to a passive backscatter because the gain of the amplifier improves the detection of the transponder.

**Figure 3:**
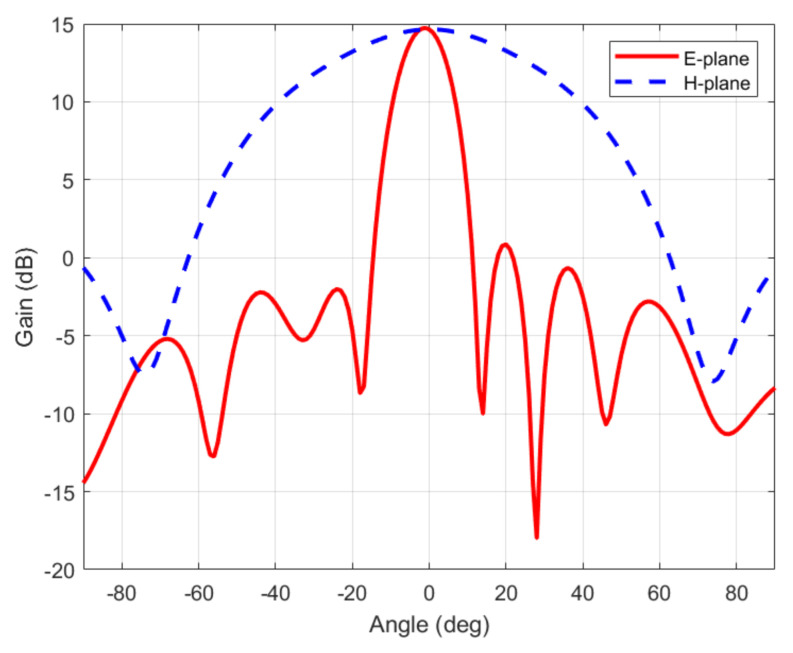
Simulation of the gain of the antenna including the cover.

**Figure 4:**
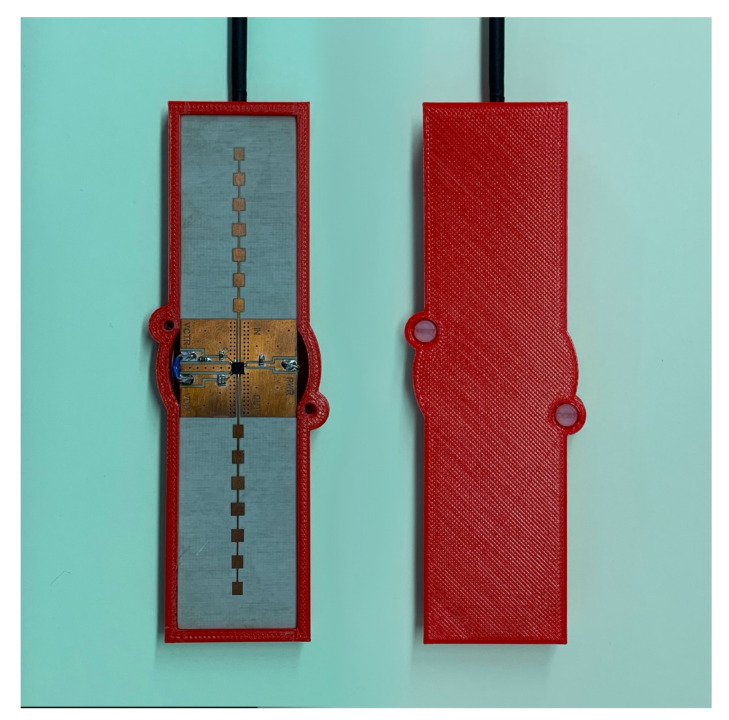
Photography of modulating transponder prototype.

To protect the circuit, a 3D printed box made with polylactic acid (PLA) has been made. The cover could introduce undesired reflections, reducing the antenna’s gain. To avoid this unwanted effect while preserving protection, a simple radome design based on a half-wave-thick PLA layer (3.9 mm) is considered [31]. PLA at millimeter band was characterized at 24 GHz in [32], obtaining a dielectric constant of 2.55 and a dissipation factor of 0.02. The reflections at each interface are canceled because they are out of phase, resulting in a high transmission coefficient. Figure 3 shows the simulation of the antenna’s gain including the cover. The array has a gain of 14.6 dBi and sidelobe levels on the order of 13.8 dB below the peak level of the main beam. From (1), the differential RCS of transponder is 12.8 dB at 24 GHz. A photograph of the transponder front end is shown in Figure 4. The dimensions of the prototype, including the protection box, are 13 cm × 3.5 cm × 1 cm.

### 2.2. Tag Detection

**Figure 5:**
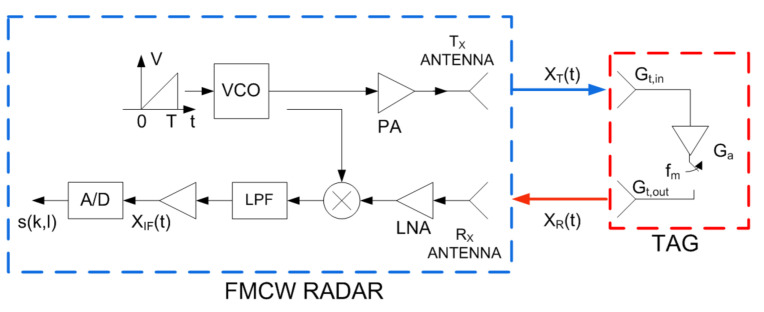
Block diagram of an FMCW with a backscatter modulated tag.

This section studies the tag detection by means of the conventional range-Doppler analysis in a FMCW radar. Figure 5 shows a block diagram of a FMCW radar. By means of the range-Doppler matrix, FMCW radars can measure the range and velocity of the targets. The transmitted signal by a FMCW radar can be expressed as a train of chirps:

(2) $$x_{T}\left(t\right)=\sum_{l=0}^{\infty }x_{c}\left(t-lT\right),$$

where $x_{c}\left(t\right)$ is a chirp function of duration *T*:

(3) $$x_{c}\left(t\right)=\exp\left(j2\pi \left(f_{c}t+\frac{1}{2}\mu t^{2}\right)\right)rect\left(\frac{t}{T}\right)$$

rect(t) denotes a rectangular pulse signal, $f_{c}$ is the carrier frequency, μ=B/T is the sweeping slope, *T* is the sweep period and $B=f_{max}-f_{min}$ is the transmission bandwidth.

The tag reflects the incoming wave from the radar. Therefore, the received signal at the radar receiver is attenuated due to the propagation distance and is delayed by the round trip delay time τ=2r/c, where *c* is the light velocity and *r* is the distance between the tag and the radar:

(4) $$r=r_{0}+vt,$$

being $r_{0}$ the initial distance between the tag and the radar and *v* the radial relative velocity between both. The tag modulates the radar signal multiplying it by a modulation pulse p(t) corresponding to the switching waveform applied to the power supply of the amplifier. This function can be approximated by a rectangular waveform. The signal received at the tag can be expressed as:

(5) $$x_{R}\left(t\right)=A\sum_{l=0}^{\infty }x_{c}\left(t-lT-\tau \right)\sqrt{\sigma }p\left(t\right),$$

where *A* takes into account the propagation attenuation and σ is the radar cross section (RCS) associated with the structural mode of the tag. The modulation pulse p(t) can be approximated by a rectangular waveform with a 50% duty cycle:

(6) $$p\left(t\right)=1+\Delta \sum_{l=1}^{\infty }rect\left(\frac{t-lT}{T_{m}/2}\right),$$

where Δ is the difference between the backscattered fields of both switching states and $T_{m}$ is the modulation period (equal to the inverse of switching rate $f_{m}$). As p(t) is a periodic function, therefore can be expanded in a Fourier series:

(7) $$p\left(t\right)=\sum_{n=-\infty }^{\infty }c_{n}e^{j2\pi nf_{m}t},$$

where $c_{n}$ are the Fourier coefficients. In practice, these coefficients decrease quickly with the harmonic index *n*, and therefore only the first coefficients are considered because the rest fall below the noise floor. In an FMCW radar, the delay associated with τ results in a shift in the frequency of the received signal given by μτ. The received signal is mixed with the transmission signal $x_{T}\left(t\right)$. The complex beat signal at the output of the mixer after low-pass filtering is given by:

(8) $$\begin{array}{c} x_{IF}\left(t\right)=x_{R}\left(t\right)\;\cdot \;x_{T}^{*}\left(t\right)=\left(\sum_{l=0}^{\infty }y\left(t-lT\right)\right)\cdot \sum_{n=-\infty }^{\infty }c_{n}e^{j2\pi nf_{m}t}, \end{array}$$

with

(9) $$y\left(t\right)=A^{\prime }rect\left(\frac{t}{T}\right)e^{-j2\pi \left(f_{c}t-\frac{\mu \tau^{2}}{2}\right)}e^{-j2\pi \mu \tau t}.$$

The delay can be approximated by the delay at the start of each sweep ramp $\tau =2r/c=\tau_{0}+2v/c\left(lT\right)$ and neglecting the quadratic terms that are a reasonably approximation for typical values of μ and *v* [24,33,34]:

(10) $$y\left(t\right)\approx A^{\prime }rect\left(\frac{t}{T}\right)e^{-j2\pi \left(f_{c}t_{0}-\frac{\mu \tau_{0}^{2}}{2}\right)}e^{-j2\pi \mu \tau_{0}t}e^{j2\pi f_{D}lT}.$$

In (9) and (10), *A*’ is the amplitude of the receiver signal at the IF-output that takes into account the conversion gain in the mixer, the IF amplifiers, and filters (see Figure 5). Due to the car movement, the receiver signal undergoes a Doppler frequency shift $f_{D}$ given by [34,35]:

(11) $$f_{D}=-2vf_{c}/c.$$

It should be taken into account that, while either of the two cars (the one equipped with the radar or with the tag) is moving, a Doppler shift will occur, *v* being the relative velocity between them. The sign of the Doppler shift indicates the direction of the relative motion.

Modern FMCW radars are based on a digital receiver, where the received IF signal is digitalized by the ADC with sampling frequency $f_{s}$. The samples are saved into a matrix s(k,l), k=0⋯N−1, l=0⋯L−1 [24].

(12) $$s\left(k,l\right)=A^{\prime }rec\left(\frac{kT_{s}}{T}\right)\cdot \sum_{n=-\infty }^{\infty }c_{n}e^{-j2\pi \mu \tau_{0}kT_{s}}e^{j2\pi f_{D}lT}e^{j2\pi nf_{m}\left(kT_{s}+lT\right)}.$$

The target detection in automotive FMCW radars is performed from the range-frequency map (RD). It is computed from two dimensional FFT of a frame s(k,l) which is composed by *L* chirps with *N* samples by chirp [34]. The zero-padding technique is used to improve the resolution from a finite number of samples and windowing allows reducing the interference of the side lobes associated with the Fourier transform.

(13) $$RD=FFT_{doppler}\left(FFT_{range}\left(s\cdot w_{range}\right)\cdot w_{doppler}\right),$$

where $FFT_{range}$ and $FFT_{doppler}$ denote the FFT along the range index *k* and the Doppler index *l* and $w_{range}$ and $w_{doppler}$ are the windows used in range and Doppler, respectively. In this work, a Hann window (Hanning window) which is one of the most popular windows in automotive radar signal processing is chosen [36].

**Figure 6:**
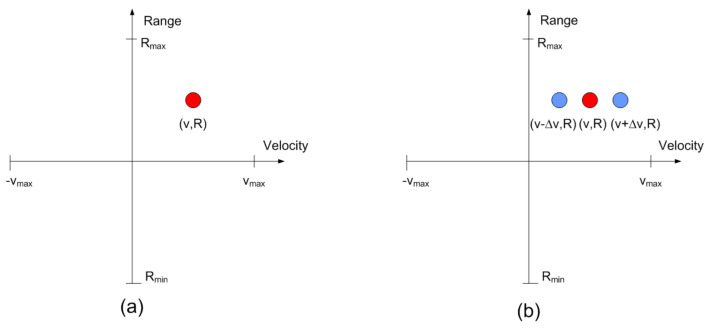
Transponder detection based on its spectral signature: (**a**) Target or transponder with the modulation off, and (**b**) modulated transponder with the modulation activated.

Automotive FMCW radar uses fast ramp chirps, therefore, for typical values of $\mu \tau >>f_{m}$. Figure 6 shows schematically the response of a modulated transponder. It is centered at the point corresponding to the range and the relative velocity of the transponder (car). The amplitude at point (R,v) is proportional to the structural mode and is overlaid with the response of the objects where the transponder is attached (e.g., the car). However, unlike a passive target, several peaks in the Doppler direction corresponding to the harmonics of the modulating waveform can be observed. Therefore, peaks are expected at velocities: $v+n\cdot f_{m}\cdot \lambda /2$, where *n* is the harmonic index and λ is the wavelength at the center frequency (see Figure 6). As the Fourier coefficients decrease very fast with the harmonic index, the higher harmonics (*n* > 1) are often below the noise background and they will not be detected. These peaks in the range-Doppler map associated with the backscatter are in the same range resolution cell. However, the velocity difference between two consecutive peaks is a function of the modulation frequency and does not depend on the relative velocity between the backscatter and the radar. Therefore, stationary or backscatter at different velocities can be detected by searching the points that comply with the last condition. A tolerance in velocity has been considered when the velocity difference between peaks is computed to prevent the influence of vibrations that can introduce some random velocity components. The average tag velocity can be calculated from the average between two consecutive peaks in the RD map.

Notice that aliasing in velocity can occur when the modulation frequency, or the car velocity, is too high. This situation happens when the velocity coordinate of the point exceeds $\pm v_{max}=\pm \lambda /\left(4T\right)$ and, consequently, alias points in the range-Doppler map are observed. These ghost targets can be classified based on the known difference in velocity.

**Figure 7:**
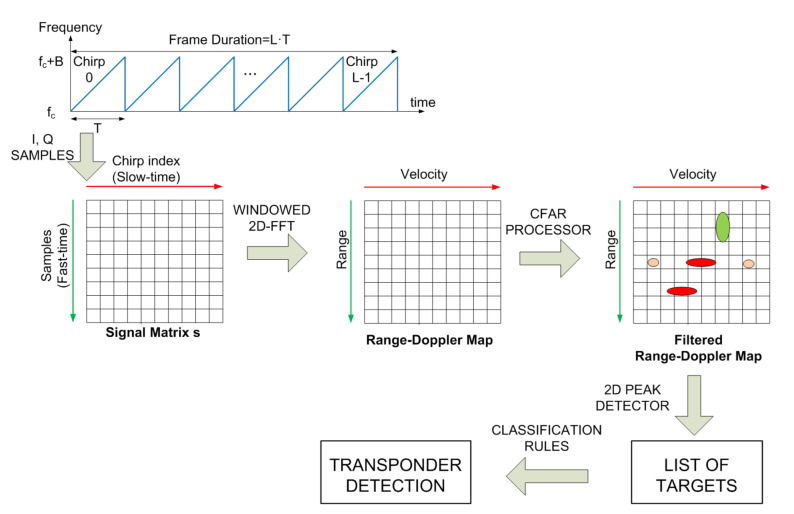
Range-Doppler matrix computation from the IQ samples and flow-chart of the signal processing.

The flow-chart of the signal processing is schematically shown in Figure 7. Before starting the classification process to detect the presence of the transponder, interference caused by noise and clutter must be removed from the range-Doppler map to avoid false alarms. The simplest method consists of removing the points that are below a constant threshold level. However, this threshold is difficult to be determined in a real environment. Therefore, an adaptive method to determine the threshold based on a constant false alarm method (CFAR) is commonly used. Different two-dimensional CFAR processors have been proposed in the literature such as constant false alarm rate (CA-CFAR) [37] or the ordered statistic constant false alarm rate (OS-CFAR) [38]. The CA-CFAR noise estimation is obtained by averaging the values of the cells in the training area [37]. Although OS-CFAR is more robust in multitarget environments [38], it requires a higher computational cost. Therefore, several alternatives have been investigated [39].

## 3. Results

**Figure 8:**
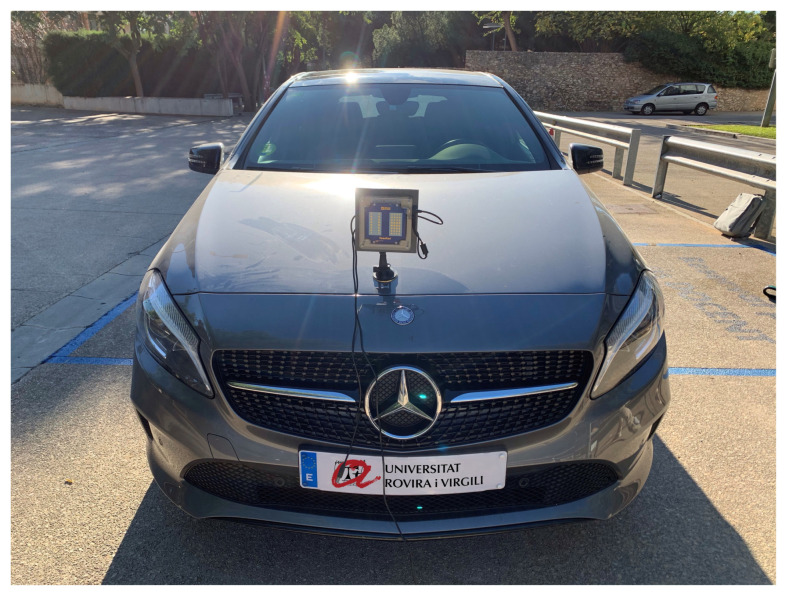
Photography of the car with the radar mounted.

**Figure 9:**
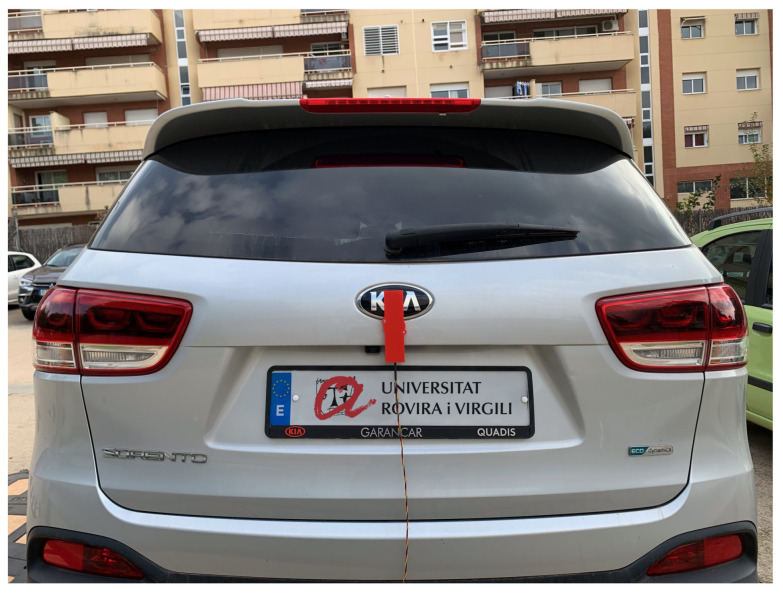
Photography of a car with the transponder installed on the back.

In order to show the feasibility of performing communication between the transponder and the radar, this section presents some experimental results. The experiment has been carried out outdoors with real vehicles but for the sake of safety, the scenario is the parking area of the University. The experiment makes use of the designed transponder and a commercial radar. EVAL-DEMORAD from Analog Devices Inc. (Norwood, MA, USA) is used to perform the measurements in the experiment. The radar is composed of two transmitters and 4 receiver antennas and is based on the 2-channel FMCW transmitter ADF5901 and the 4-channel receiver ADF5904, both from Analog Devices. The radar uses a patch antenna array design that achieves a field of view (FOV) of approximately 120 degrees in azimuth and 15 degrees in elevation. The transmitter power is 8 dBm.The FMCW radar was configured to sweep within the ISM frequency band (24–24.3 GHz) using a sampling frequency of 1.2 MHz, and the sweep time used was 250 μs. A total of 128 chirps with 256 points per chirp was used to obtain the range-Doppler maps. The radar is mounted in a car with reader role (the Benz in Figure 8) and a transponder was installed in the back of the car (The KIA in Figure 9). In the experiments, different modulation frequencies have been programmed to indicate different driving events for classification purposes.

**Figure 10:**
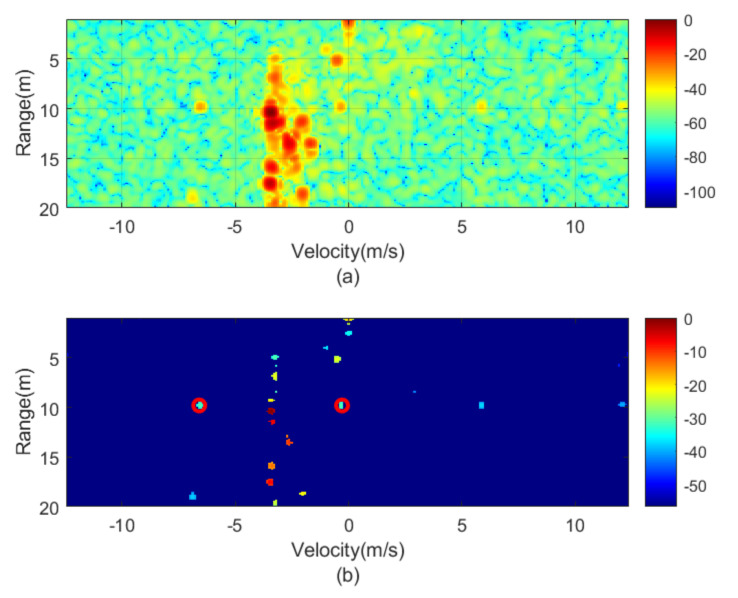
Measured range—Doppler map (**a**) and detection after CFAR processor (**b**) for the transponder modulated at 500 Hz.

**Figure 11:**
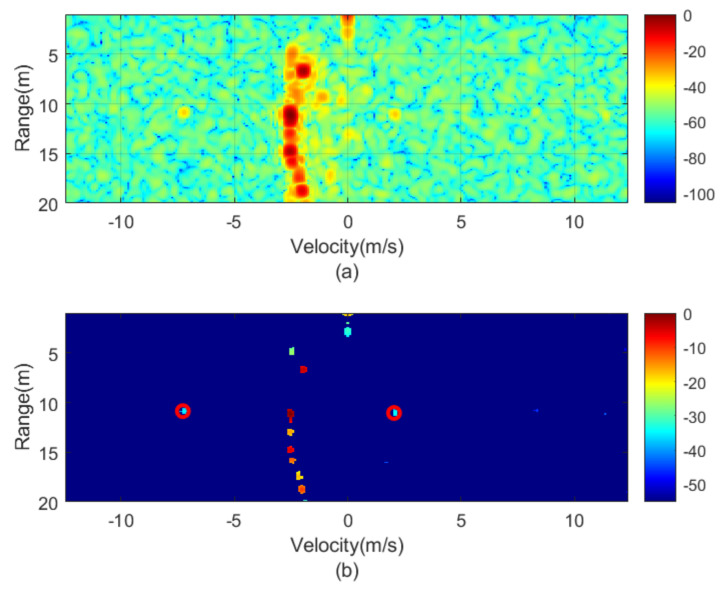
Measured range—Doppler map (**a**) and detection after CFAR processor (**b**) for the transponder modulated at 750 Hz.

**Figure 12:**
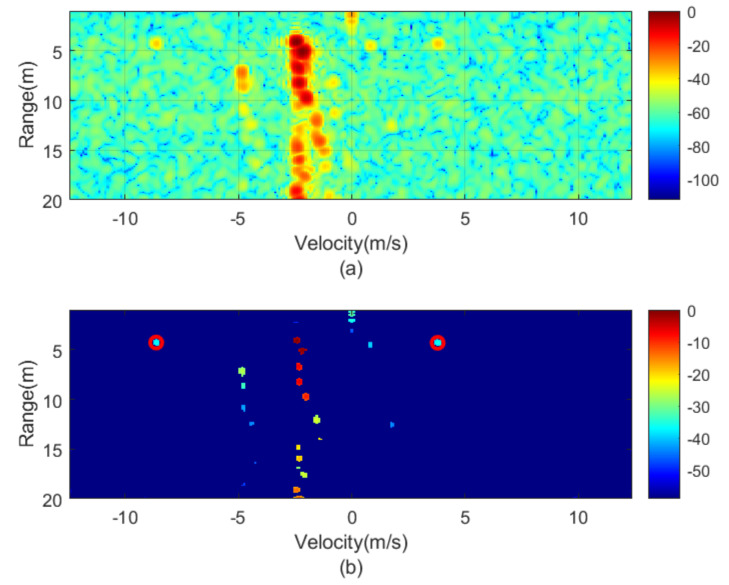
Measured range—Doppler map (**a**) and detection after CFAR processor (**b**) for the transponder modulated at 1000 Hz for a distance of about 5 m.

**Figure 13:**
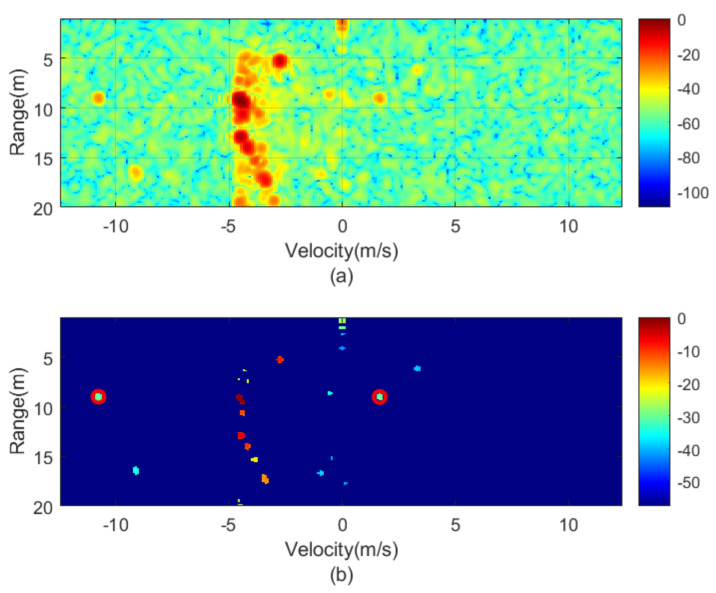
Measured range—Doppler map (**a**) and detection after CFAR processor (**b**) for the transponder modulated at 1000 Hz for a distance of about 10 m.

**Figure 14:**
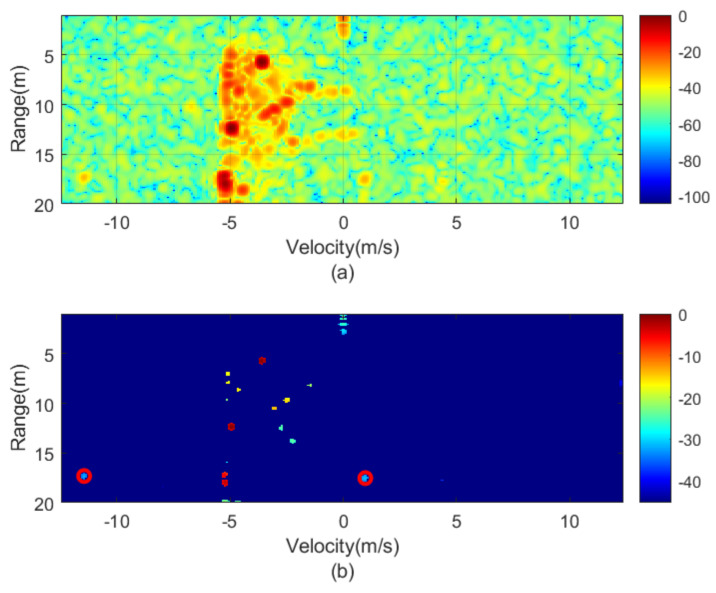
Measured range—Doppler map (**a**) and detection after CFAR processor (**b**) for the transponder modulated at 1000 Hz for a distance of about 18 m.

**Figure 15:**
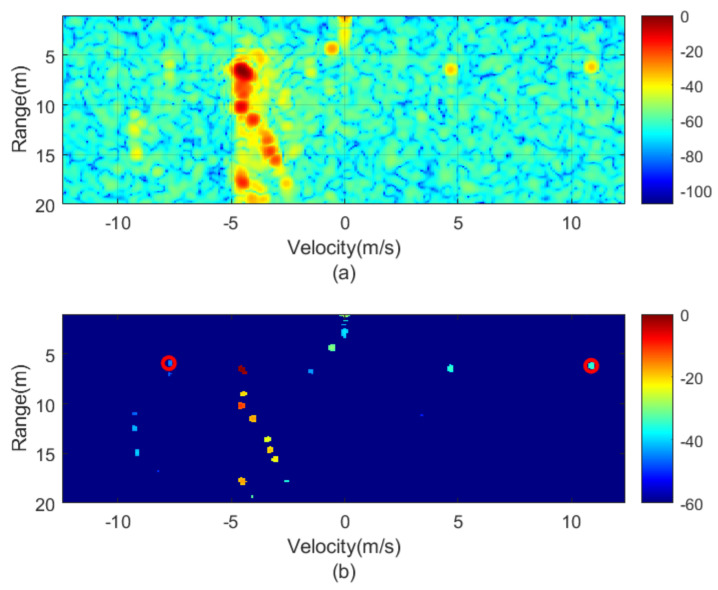
Measured range—Doppler map (**a**) and detection after CFAR processor (**b**) for the transponder modulated at 1500 Hz.

The next figures (Figure 10, Figure 11, Figure 12, Figure 13, Figure 14 and Figure 15) show the measured range-Doppler map and the output of the CFAR processor. Figure 10 and Figure 11 show measurements for $f_{m}$ equal to 500 Hz and 750 Hz, respectively. Figure 12, Figure 13 and Figure 14 show measurements at $f_{m}$ equal to 500 Hz for different distances between car from 5 m to 18 m. Figure 15 shows another measurement for $f_{m}$ = 1500 Hz at 10 m, where the effect of aliasing is visible. The conventional CA-CFAR algorithm was applied to suppress the clutter. The threshold is set at 10 dB relative to the noise estimated from the output of the CA-CFAR algorithm. In this work, 10 averaging cells and 5 guard cells on both direction axis, range and velocity, have been considered. The classification rules have been applied to automatically detect the presence of the transponder. The detected peaks associated with the transponder were encircled with a red circle. There is a detection rate of 97.42% and an average error in the measured modulation frequency of 0.5%.

**Figure 16:**
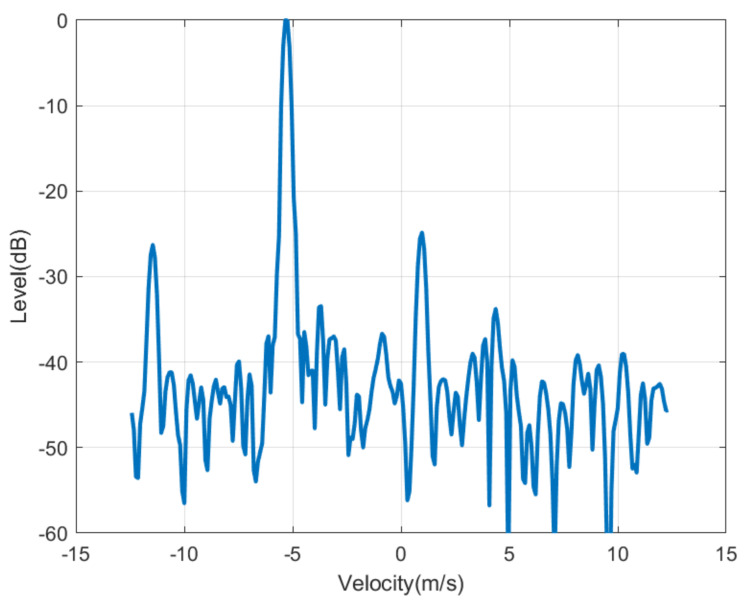
Cut of the range—Doppler map of Figure 14 as a function of the velocity for the range where is detected the transponder modulated at 1000 Hz.

Figure 16 shows a horizontal cut of Figure 14 for the range of the modulated transponder. A transponder signal level is observed approximately 25 dB below the peak associated with reflection in the car and 15 dB above the noise floor. Therefore, the transponder can be detected up to 30 m with a signal-to-noise ratio of 5 dB. The radar cross-section of the car is a function of the model, orientation and materials, but it is typically between −10 and 18 dBsm [24] and, therefore, it is 20–30 dB higher than the differential RCS of the transponder (−12.8 dBsm). The peaks associated with reflection from the car and the transponder are shifted due to the Doppler effect caused by the relative velocity between the cars. The separation between the two peaks associated with the transponder is $2\cdot f_{m}\cdot \lambda /2=$ 12.5 m/s for $f_{m}=$ 1000 Hz and it agrees with the theory. The maximum unambiguous modulation frequency is limited by the maximum velocity ($v_{max}$) that the radar can be measured and therefore the sweep time *T*. However, the theoretical limit in the minimum step between the modulation frequency that the radar can resolve is a function of the velocity resolution that, at the same time, is a function of the number of chirp per frame, *L*. Considering the experiment, *L* = 128 frames. Up to 128 events can be encoded, which is more than enough for the proposed application. However, more advanced encoding schemas using multi-level Frequency Shift Keying (FSK) between several frames can be considered to send more complex messages changing the modulation frequency $f_{m}$ in the transponder.

## 4. Discussion

**Figure 17:**
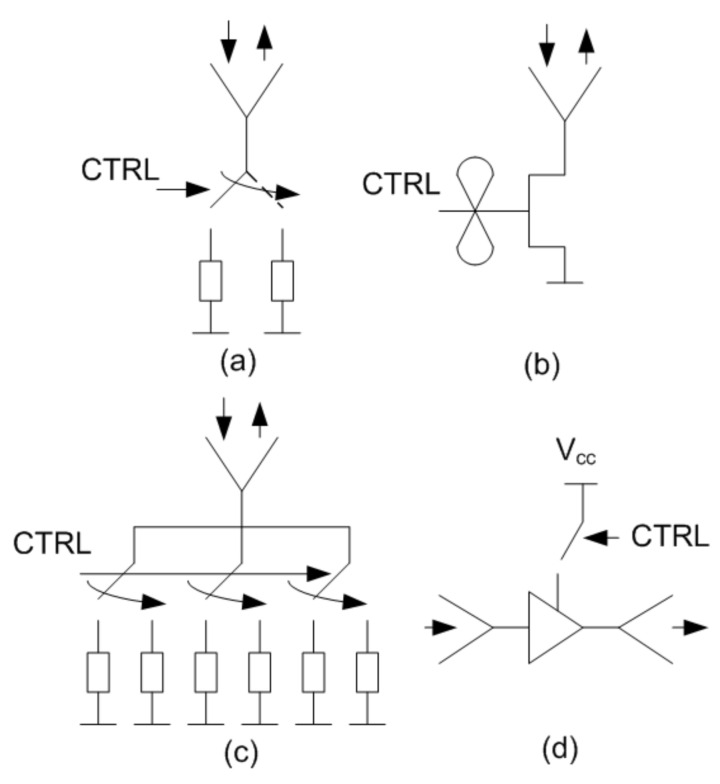
Common mm-wave backscatter topologies: (**a**) backscatter based on a switch, (**b**) backscatter based on a Cold-FET, (**c**) retrodirective backscatter and (**d**) transponder using an amplifier.

The car-to-car solution, based on the modulation transponder, can employ other topologies of backscatters. Table 1 summarizes recent works presented in the literature that use different semi-passive millimeter-wave backscatters. It can be classified according to the modulation technique and the device technology. Figure 17 shows the schemas of the most common modulation techniques used in the design of backscatters. The main challenge in the design of backscatter is the availability of devices with low parasitic components, especially when considering packaged devices, which can work properly at millimeter waves. The simplest topology, shown in Figure 17a, switches two impedances (e.g., open circuit and short circuit) connected to the antenna. This topology is commonly used in sub-6 GHz RF backscatters. Low-cost commercial CMOS switches, such as ADG902 from Analog Devices, are available, achieving low-losses [40]. However, PIN diodes or Cold-FET transistors can replace CMOS switches to design low-loss switches in mm-waves (see Figure 17b). In [6], a 76 GHz phase-modulated backscatter transponder based on a Hittite HMC-SDD112 GaAs PIN MMIC switch connected to a WR-12 waveguide transition and a horn antenna was presented. This device requires 22 mA at 5V to achieve low-conduction resistance for the ON state. An alternative is to use Cold-FET to reduce the current consumption. A Cold-FET consists of a transistor (often a HEMT transistor) with (drain-to-source bias equal to zero). When a voltage below the threshold is applied to the gate its behaviour is that of an open circuit (only limited by parasitic capacitance and electrode parasitics), whereas for higher voltages it works as a controlled resistance that depends on the gate voltage [41]. Low-current consumption backscatter can be designed with this solution even if packaged transistors are used. In [8], a die E-PHEMT from Avago tech was used to design a 24 GHz modulated backscatter using the topology of Figure 17b. In [7], a modulator based on an MMIC BiCMOS Cold-FET is recently presented. Packaged PHEMT transistors CE3520K3 from CEL (California Eastern Laboratories) in Cold-FET configuration were used to modulate a Rotman-lens-based retrodirective array achieving a differential RCS of −15.4 dBsm at 28.5 GHz with a variation of 8 dB from −60° to 60° of interrogation angle. The differential RCS of a passive backscatter can be computed from 1 but setting the amplifier gain to unity.

To achieve high differential RCS values high gain antennas and low-loss devices (to reduce the losses in the modulation factor) are required. However, high gain antennas are too directive for some applications where high interrogation angles are needed. One solution, such as that proposed in [9], is the utilization of retrodirective arrays (see Figure 17c).

In this work, due to the use of an amplifier (see topology Figure 17d), high RCS differential values can be achieved with moderately low gain antennas (e.g., patch arrays) simplifying the size and complexity of the design compared to retrodirective arrays (e.g., Rotman lenses or Van Atta arrays). The availability of low-cost and low-power consumption (<6 mA at 3 V in our design) MMIC packaged amplifiers (<6 mA at 3 V in our design) may allow for the future commercialization of these transponders. Modulated backscatters, such as those proposed in this work, can be used to distinguish vehicles from sensitive road elements, such as pedestrian or bikes, since the latter can be masked by the presence of large objects such as other parked vehicles with higher RCS. Pedestrian or bikes equipped with these tags can be seen with a characteristic radar signature whose presence can be detected by the radar on the vehicles. Besides, modulated backscatters can help measure vital signs (e.g., breathing rate) using radars (Doppler or FMCW [42,43] or UWB [44]) that are interfered with by the body movement determining the range and velocity of the body.

## 5. Conclusions

This paper has studied the feasibility of using modulated backscatter tags for car2car communications over an onboard ADAS FMCW radar playing a reader role. For safety purposes, introducing redundancy to the detection of this warning information is under consideration for autonomous driving but also for safer driving. The objective pursued is the reduction of collisions due to driver distractions or lack of visibility caused by weather conditions. A proof-of-concept transponder at 24 GHz has been designed. It is based on a low-power consumption SiGe MMIC amplifier connected between two series feed patch antenna arrays. The utilization of an active device with gain allows the improvement of the detectability of the transponder. The modulation was performed by switching the DC feed power connected to a low-power microcontroller that can change the modulation frequency for classification purposes depending on the event to be identified (e.g., turn lights, stop, back direction). Each event is encoded with a different modulation frequency that can be demodulated in the radar. The modulation of the RCS allows the detection of the transponder in the presence of strong unmodulated backscatters such as the metallic car body itself. A simple detection algorithm for the automatic detection of the transponder state from the measured range-Doppler map has been proposed. It is based on the classification of the list of targets given by the standard automotive signal processing at the output of the CFAR processor. Peaks spaced in Doppler by two times the modulation frequency allow for the detection of the transponder. Therefore, the system does not require hardware modifications and it is compatible with the on-board automotive radar software.

## Figures and Tables

**Table 1:** Millimeter band backscatters.

Ref.	Technology	Frequency	Application
[6]	Pin diode	77 GHz	FMCW radar calibration
[8]	Cold FET (E-PHEMT)	24 GHz	Communications
[7]	Cold FET (BiCMOS)	24 GHz	5G-IoT
[9]	Cold FET (PHEMT), Retrodirective Rotman lens antenna array	28 GHz	Long-range MMID
This work	SiGe amplifier	24 GHz	Automotive car2car

## Data Availability

The data presented in this study are available on request from the corresponding author.
